# Eccentric Exercise Facilitates Mesenchymal Stem Cell Appearance in Skeletal Muscle

**DOI:** 10.1371/journal.pone.0029760

**Published:** 2012-01-11

**Authors:** M. Carmen Valero, Heather D. Huntsman, Jianming Liu, Kai Zou, Marni D. Boppart

**Affiliations:** Department of Kinesiology and Community Health, and Beckman Institute for Advanced Science and Technology, University of Illinois, Urbana, Illinois, United States of America; McMaster University, Canada

## Abstract

Eccentric, or lengthening, contractions result in injury and subsequently stimulate the activation and proliferation of satellite stem cells which are important for skeletal muscle regeneration. The discovery of alternative myogenic progenitors in skeletal muscle raises the question as to whether stem cells other than satellite cells accumulate in muscle in response to exercise and contribute to post-exercise repair and/or growth. In this study, stem cell antigen-1 (Sca-1) positive, non-hematopoetic (CD45^-^) cells were evaluated in wild type (WT) and α7 integrin transgenic (α7Tg) mouse muscle, which is resistant to injury yet liable to strain, 24 hr following a single bout of eccentric exercise. Sca-1^+^CD45^−^ stem cells were increased 2-fold in WT muscle post-exercise. The α7 integrin regulated the presence of Sca-1^+^ cells, with expansion occurring in α7Tg muscle and minimal cells present in muscle lacking the α7 integrin. Sca-1^+^CD45^−^ cells isolated from α7Tg muscle following exercise were characterized as mesenchymal-like stem cells (mMSCs), predominantly pericytes. *In vitro* multiaxial strain upregulated mMSC stem cells markers in the presence of laminin, but not gelatin, identifying a potential mechanistic basis for the accumulation of these cells in muscle following exercise. Transplantation of DiI-labeled mMSCs into WT muscle increased Pax7^+^ cells and facilitated formation of eMHC^+^DiI^−^ fibers. This study provides the first demonstration that mMSCs rapidly appear in skeletal muscle in an α7 integrin dependent manner post-exercise, revealing an early event that may be necessary for effective repair and/or growth following exercise. The results from this study also support a role for the α7 integrin and/or mMSCs in molecular- and cellular-based therapeutic strategies that can effectively combat disuse muscle atrophy.

## Introduction

It is well accepted that maintenance of skeletal muscle mass plays a critical role in overall health and quality of life. The slow decline in contractile protein content and subsequent reduction in muscle fiber size with disease, immobility, or age can have detrimental consequences not only to strength, but also metabolic disease risk, including susceptibility to obesity, diabetes, and cardiovascular disease. Engagement in physical activity or rehabilitation therapy can preserve muscle mass and function. Therefore, identification of the critical mechanisms that underlie beneficial adaptations to physical activity can be informative in the development of effective molecular- or cell-based therapies.

Skeletal muscle regeneration in response to injury has traditionally been attributed solely to the satellite cell population, adult stem cells which lie between the sarcolemma and the basal lamina and become activated, proliferate, migrate and engraft as myoblasts at sites of damage within myofibers. However, a variety of mesenchymal-like stem cells (MSCs) residing in skeletal muscle also contribute to repair in response to damage [1]–[4]. These mononuclear cells have been isolated and categorized as multipotent muscle-derived stem cells (MDSC) [5], [6], side population (SP) cells [4], [7]–[9], muscle resident progenitor cells [10], mesoangioblasts [2] and pericytes [3] based on method of extraction, localization within muscle, cellular function, and heterogeneous cell surface markers. Despite the inability to clearly and efficiently distinguish among the different adult stem cell populations, stem cell antigen-1 (Sca-1)/lymphocyte antigen 6 (Ly-6A) is a commonly expressed murine glycosyl phosphatidylinositol-anchored cell surface protein that is used in combination with other cell surface markers to identify MSCs in muscle. The majority of isolated Sca-1^+^ MSCs do not express myogenic markers (Pax7, MyoD) and vary in their ability to spontaneously differentiate into skeletal muscle [1], [3], [9]–[11], yet can readily fuse with myoblasts in co-cultures and/or can secrete factors that potently activate satellite cells in response to injury or disease [1], [3], [4], [10], [12].

Eccentric lengthening contractions result in muscle strain and subsequent injury, initiating satellite cell proliferation, new fiber synthesis, and positive adaptations in non-diseased tissues that enhance muscle fiber size and force production with training [13], [14]. A role for satellite cells in exercise-induced growth is largely debated [13], [15]–[18], however, new fiber synthesis and myonuclei addition have been demonstrated in human skeletal muscle following exercise or overload [16], [18], [19]. Likewise, the extent to which non-satellite stem cells, including Sca-1^+^ MSCs, appear in muscle and contribute to post-exercise adaptations is not known. To our knowledge, only three studies have demonstrated non-satellite stem cell accumulation and/or engraftment in skeletal muscle in response to exercise and all three focused on bone marrow-derived stem cells (BMDCs), identified by florescent labeling of transplanted cells or the combined presence of Sca-1 and CD45, a hematopoietic stem cell marker. LaBarge *et al.* demonstrated that BMDCs are enhanced in the satellite cell niche following six months of voluntary wheel exercise, yet total engraftment was only 3.5% [20]. More efficient engraftment occurred with repeated bouts of forced downhill running exercise on a treadmill (1 hr, 3 times/wk, 1 or 4 weeks) [21]. A separate study did not observe an increase in Sca-1^+^CD45^+^ cells following downhill running, despite a 6-fold increase in Myf5-expressing cells in the muscle [22]. These studies suggest that eccentric contractions may stimulate recruitment of BMDCs to muscle, but the extent of migration and engraftment may be limited. Thus, we felt attention should be directed toward examining the presence of muscle resident Sca-1^+^CD45^−^ cells following eccentric exercise and the extent to which these cells influence post-exercise myogenesis.

Integrins are heterodimers comprised of non-covalently bound α and β subunits. Integrins transverse cellular membranes and adhere extracellular matrix to the cytoskeletal network, providing a mechanism for cells to sense and respond to mechanical and chemical signals within the environment [23]. The α7β1 integrin is enriched in specialized areas of skeletal muscle [24]–[26] and its importance in muscle is clearly evident in humans and murine models that progressively develop myopathy when the α7 integrin gene is mutated or the protein is not present [27], [28]. We have previously reported that α7 integrin RNA and total protein increase in response to eccentric exercise in the form of downhill running, and muscle-specific transgenic expression of the α7BX2 integrin protects against exercise-induced muscle damage [29], [30]. Our most recent results suggest that macrophage accumulation is markedly suppressed and maximal isometric force is preserved in α7 integrin transgenic mice (α7Tg) following exercise, likely contributing to a phenotype of enhanced growth, including new fiber synthesis and marked fiber hypertrophy [31], [32]. Thus, the primary role for increased endogenous α7 integrin expression post-exercise may be to protect injured muscle from further damage upon subsequent contractile events and simultaneously facilitate muscle repair and/or growth.

The primary purpose of this study was to determine whether eccentric exercise stimulates the appearance of Sca-1^+^CD45^−^ non-satellite stem cells in skeletal muscle and the extent to which injury is necessary for this event. We hypothesized based on our previous findings that Sca-1^+^CD45^−^ cells would increase in muscle following exercise and that these cells would expand in a muscle environment resistant to injury (α7Tg). This study demonstrates that Sca-1^+^CD45^−^ stem cells, predominantly characterized as pericytes, accumulate in skeletal muscle in an α7 integrin-dependent manner and significantly contribute to satellite cell expansion and new fiber synthesis following acute eccentric exercise. In addition, we conducted *in vitro* experiments to reveal a cooperative role for mechanical strain and extracellular matrix in dictating Sca-1^+^CD45^−^ stem cell fate.

## Results

### Sca-1^+^ Cells Reside in Connective Tissue and Vascular Dense Areas in Skeletal Muscle

**Figure 1:**
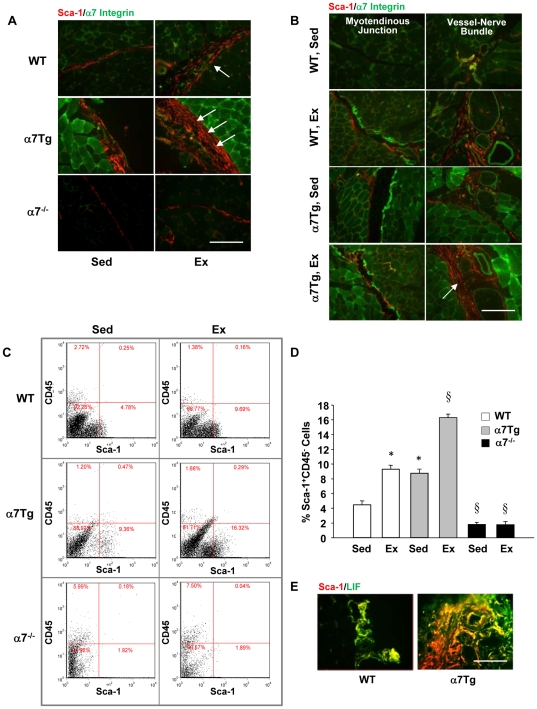
The α7 integrin is prerequisite for Sca-1^+^ stem cell accumulation in skeletal muscle following exercise. (*A*) Localization of stem cell antigen-1 (Sca-1) (arrows, TRITC-red) positive mononuclear cells and α7 integrin (FITC-green) in the interstitium of wild type (WT), α7 transgenic (α7Tg), and α7^−/−^ skeletal muscle in the sedentary state (Sed) or 24 hr post-exercise (Ex) at 20×. (*B*) Localization of Sca-1 (TRITC-red) positive mononuclear cells and α7 integrin (FITC-green) in the myotendinous junction and in close proximity to vessels and nerves of WT and α7Tg skeletal muscle in the sedentary state (Sed) or post-exercise (Ex). (*C*) FACS analysis of Sca-1 and CD45 expression on mononuclear cells extracted from skeletal muscle in the sedentary state and post-exercise. (*D*) Quantitation of Sca-1^+^CD45^−^ mononuclear cells in the sedentary state (Sed) and 24 hr post-exercise (Ex). (E) Representative images showing co-localization of Sca-1^+^ cells with leukemia inhibitory factor (LIF) in the vascular niche of WT and α7Tg muscle. Scale bar = 10 µm. Data are means ± SEM, n = 4–6/group for *C* and *D*. * P<0.05 vs. WT Sed and § P<0.05 vs. all groups.

Inflammation can be a potent chemoattractant for stem cell migration in skeletal muscle [33]. The resistance to exercise-induced damage and inflammation in α7Tg mouse muscle prompted us to question whether injury was prerequisite for Sca-1^+^ stem cell appearance following eccentric exercise. Sca-1^+^ cell localization was examined in samples previously used to characterize the injury response to eccentric exercise in WT, α7Tg, and α7^−/−^ muscle (29,30). Important structures such as the sarcolemma, myotendinous junction and vessels were identified by co-staining with α7B integrin. Figure 1A shows the accumulation of Sca-1^+^ cells in the connective tissue (interstitium) surrounding bundles of fibers following exercise in WT, with greater cell density observed in α7Tg. In contrast, minimal Sca-1^+^ cell staining was observed in α7^−/−^ muscle. Sca-1^+^ cells also resided around vessels and the myotendinous junctions to a greater degree in α7Tg compared to WT muscle, both in the rested state and post-exercise (Fig. 1B). Expression in single cells was verified using a nuclear stain and visualization at higher magnification (63×) (Fig. S1A). Sca-1 expression was observed in the membrane of mononuclear cells in the interstitium, but co-localization of Sca-1 with α7 integrin within single cells was rare.

### The α7 Integrin Regulates the Appearance of Sca-1^+^CD45^−^ Cells in Skeletal Muscle Following Eccentric Exercise

The Sca-1 and CD45 profile of total mononuclear cells extracted from WT, α7Tg, and α7^−/−^ skeletal muscle was evaluated by fluorescence-activated cell sorting (FACS) (Fig. 1C). Whereas no differences were noted in the percentage of Sca-1^−^CD45^−^ and Sca-1^+^CD45^+^ cells in any of the groups (Table S1), a significant difference was detected between group and treatment (P<0.001) in the percentage of Sca-1^+^CD45^−^ cells. Sca-1^+^CD45^−^ cells were increased 2.2-fold in WT mice post-exercise compared to WT mice that did not run, and the presence of the α7 integrin transgene alone also increased Sca-1^+^CD45^−^ cells to the same extent (P<0.05) (Fig. 1D). In α7 transgenic mice, 16.2% of the mononuclear fraction was Sca-1^+^CD45^−^ following exercise, a 3.8-fold increase over WT mice in the sedentary state. This cell fraction is infrequently observed in α7^−/−^ mice either in the rested state or post-exercise, suggesting the presence of the α7 integrin is an essential regulator of resident stem cells in muscle (Fig. 1D). The percentage of Sca-1^−^CD45^+^ cells, presumably inflammatory cells, was increased in α7^−/−^ mice which are characterized by progressive muscle disease and susceptibility to exercise-induced damage (Table S1). Leukemia inhibitory factor (LIF) expression is a predictor of early progenitor status in stromal cells [34]. LIF expression co-localized with Sca-1^+^ cells in the vascular niche of WT and α7Tg muscle (Fig. 1E). LIF was not present in α7^−/−^ muscle concomitant with lack of Sca-1^+^ cells (data not shown).

### Sca-1^+^CD45^−^ Cells Isolated from α7Tg Muscle Post-exercise are Mesenchymal-like Stem Cells, Predominantly Pericytes

**Figure 2:**
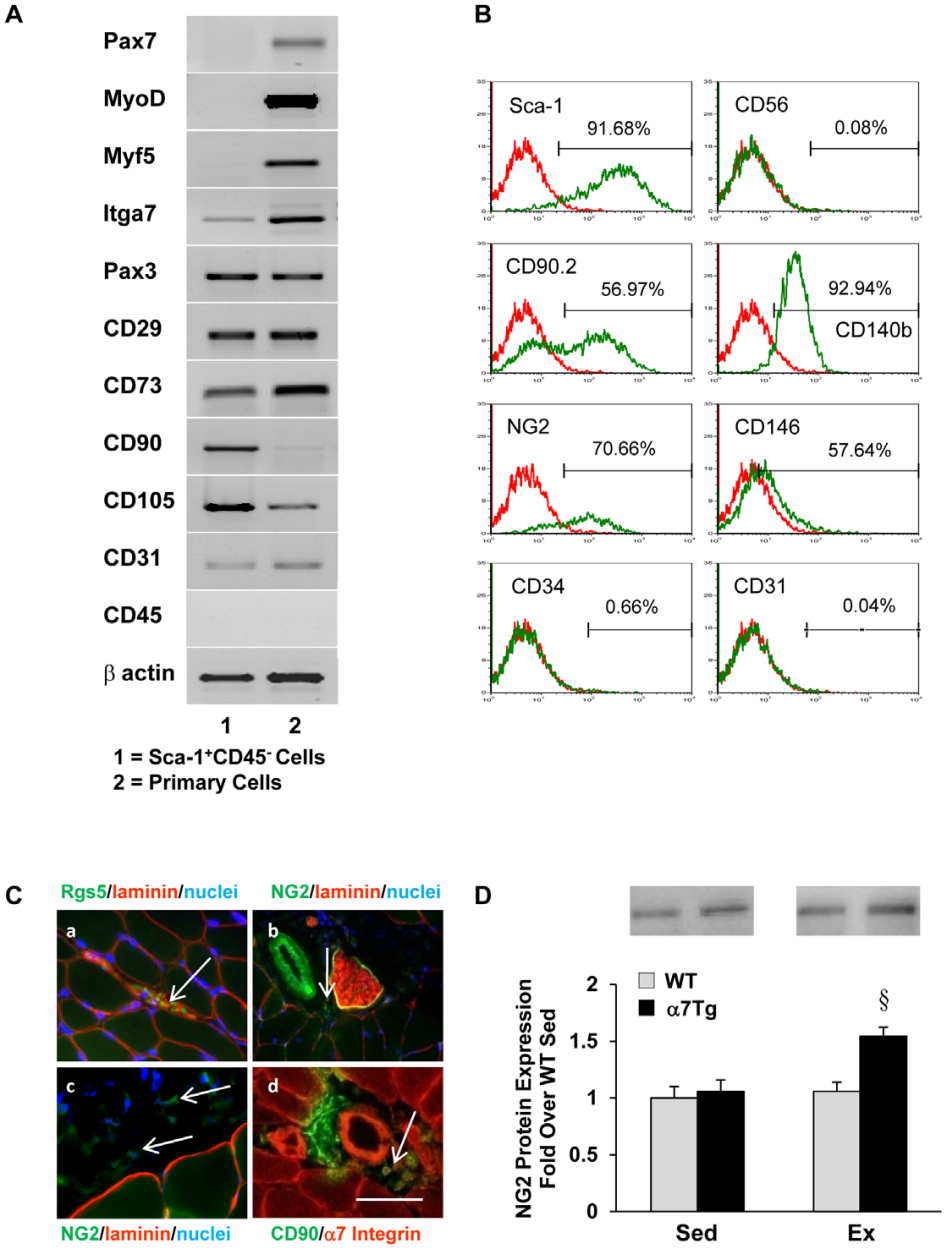
Sca-1^+^CD45^−^ cells extracted from α7Tg skeletal muscle post-exercise are multipotent mesenchymal-like stem cells, predominantly pericytes. (*A*) RT-PCR analysis of RNA transcripts from Sca-1^+^CD45^−^ cells extracted from α7Tg muscle post-exercise and primary muscle control cells. (*B*) Flow cytometry analysis of cell surface markers from Sca-1^+^CD45^−^ cells extracted from α7Tg muscle 24 hr post-exercise. (*C*) Localization of (a) Rgs5^+^ mononuclear cells (arrows, FITC-green) in the interstitium at 40×; (b) NG2^+^ mononuclear cells (arrow, FITC-green) in close proximity to vessels and nerves at 40×; (c) NG2^+^ mononuclear cells (arrows, FITC-green) in the interstitium; (d) CD90^+^ mononuclear cells (arrows, FITC-green) in close proximity to vessels at 40×. (*D*) NG2 (pericyte) protein expression in skeletal muscle. Scale bar = 5 µm. Data are means ± SEM, n = 4–5/group for *D*. § P<0.05 vs. all groups.

To further characterize the Sca-1^+^CD45^−^ cells residing in α7Tg muscle post-exercise, myogenic and mesenchymal stem cell (MSC)-specific transcripts were evaluated in Sca-1^+^CD45^−^ cells extracted from α7Tg muscle and unsorted primary cells from WT muscle using RT-PCR (Fig. 2A). Whereas both primary and Sca-1^+^CD45^−^ cells similarly expressed Pax3, only primary cells expressed Pax7, MyoD and Myf5. In previous studies, Pax3 expression is upregulated in non-satellite muscle progenitors upon conversion to skeletal muscle [10] and can determine myogenic fate in bone marrow-derived MSCs [35]. Several MSC markers, including CD29, CD73, and CD105 were co-expressed by primary and Sca-1^+^CD45^−^ cells, but CD90 was restricted to Sca-1^+^CD45^−^ cells. Rgs5, a pericyte marker is increased in muscle overexpressing the α7B integrin [36]. Thus, specific markers were examined by flow cytometry to determine the extent to which pericytes comprise the Sca-1^+^CD45^−^ mononuclear cell fraction in α7Tg muscle post-exercise. Greater than 50% of the sorted cells expressed common pericyte markers, including CD140b (PDGFRβ) (93%), NG2 (71%), CD146 (58%), and CD90.2 (57%), and less than 1% expressed endothelial markers CD31 or CD34 (Fig. 2B). Localization of pericyte-specific markers was visualized in α7Tg skeletal muscle cross sections by staining for Rgs5, NG2 and CD90 and either laminin or α7B integrin to outline fibers and vessels (Fig. 2C). NG2 protein was also increased in α7Tg skeletal muscle post-exercise, confirming expansion of pericytes 24 hr post-exercise (P<0.05 α7Tg Ex vs. all other groups) (Fig. 2D). Based on CD56 labeling (0.08%), satellite cells did not comprise the Sca-1^+^CD45^−^ population extracted from muscle post-exercise (Fig. 2B). Finally, MSCs are multipotent and have the capacity to differentiate into a variety of mesodermal tissues. Sca-1^+^CD45^−^ muscle-derived mesenchymal stem cells (mMSCs) extracted from α7Tg muscle following exercise differentiated into adipocytes, chondrocytes, and osteogenic cells when exposed to appropriate media (Fig. S1B).

### Mechanical Strain and Laminin, not Proliferation, may Account for Increased Appearance of mMSCs in Muscle Post-Exercise

**Figure 3:**
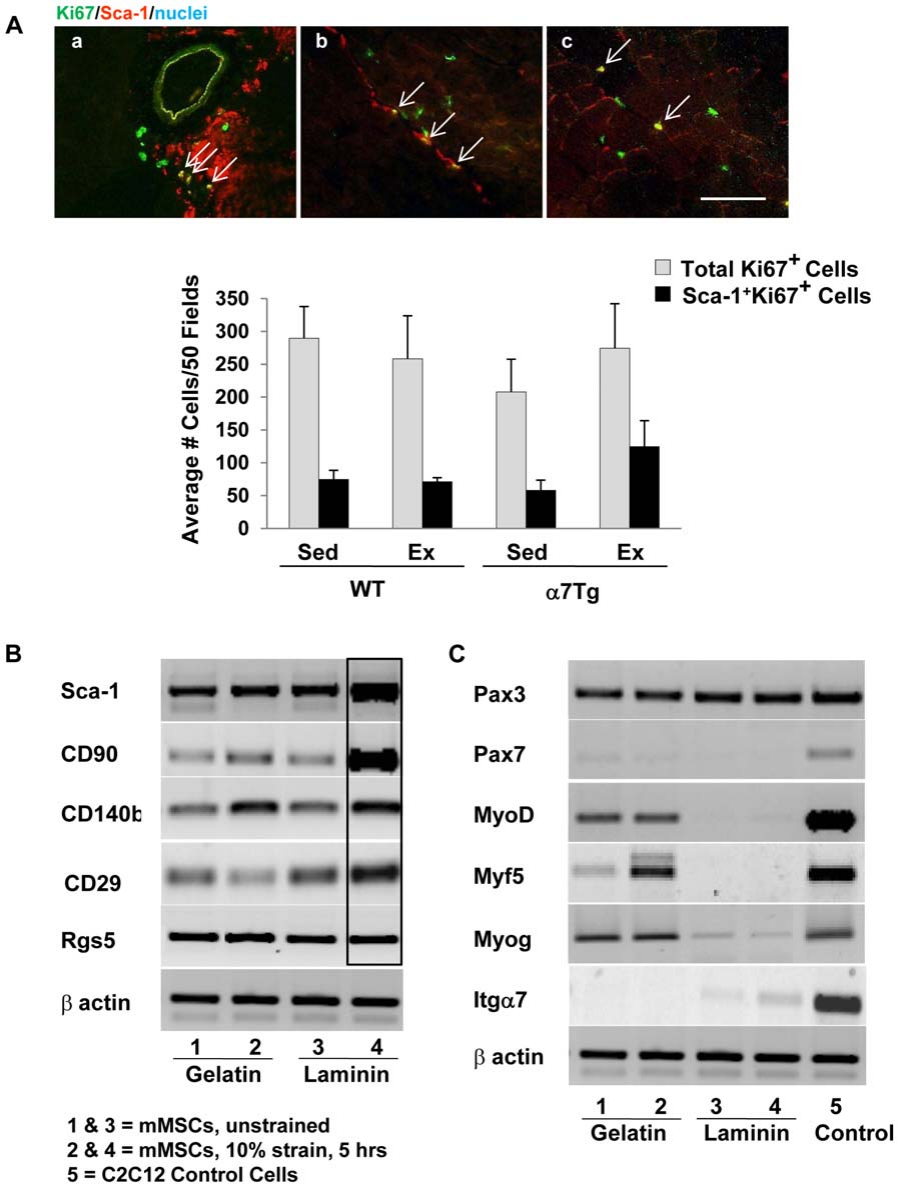
Mechanical strain and substrate, not proliferation, may account for increased mMSC appearance in skeletal muscle following exercise. (*A*) Ki67^+^ cells (FITC-green) and cells coexpressing Ki67 and Sca-1 (TRITC-red) were identified using immunofluorescence methods in skeletal muscle sections of wild type (WT) and α7Tg mice in the sedentary state (Sed) and 24 h post-exercise (Ex). Images: (a) co-staining in vessel-nerve bundle, (b) co-staining in myotendinous junction, (c) co-staining around fibers/interstitium. Total number of Ki67^+^ cells and Ki67^+^ cells expressing Sca-1 were quantified. (*B*) RT-PCR analysis of mesenchymal stem cell and pericyte RNA transcripts from mMSCs following no strain (lanes 1 and 3) or 5 hr of 10% multiaxial strain (lanes 2 and 4) in the presence of gelatin (lanes 1 and 2) or laminin (lanes 3 and 4). (*C*) RT-PCR analysis of myogenic RNA transcripts from mMSCs following no strain (lanes 1 and 3) or 5 hr of 10% multiaxial strain (lanes 2 and 4) in the presence of gelatin (lanes 1 and 2) or laminin (lanes 3 and 4). Lane 5 represents results for C2C12 cells incubated on uncoated plastic culture dishes. Scale bar = 10 µm. Data are means ± SEM, n = 4/group for *A*.

Sca-1^+^CD45^−^CD31^−^ side population cells, isolated by Hoechst 33342 dye exclusion, are highly proliferative after extraction from cardiotoxin-induced muscle damage [4]. We sought to determine whether mMSCs were more abundant in α7Tg mice following exercise as a result of enhanced proliferation. The total number of Ki67^+^ cells and the fraction of Sca-1^+^ cells expressing Ki67 were evaluated in WT and α7Tg muscle cross sections in the rested state and 24PE (Fig. 3A). Although Ki67 expression appeared higher in Sca-1^+^ mononuclear cells in α7Tg mice 24PE, significant differences were not detected between groups for either total or Sca-1^+^ mononuclear cell Ki67 staining (Fig. 3A). We examined Ki67 expression at 3 hr post-exercise in a preliminary subset of mice to address the possibility that proliferation occurred earlier than 24 hr, but did not detect a significant increase. Thus, proliferation does not appear to fully account for the accumulation of mMSCs in α7Tg muscle 24 hr following exercise.

Sca-1 expression is upregulated in activated lymphocytes [37] and myoblasts exposed to crushed muscle extract [38]. Thus, Sca-1 appears to be dynamically regulated by a variety of factors. To address whether forces associated with muscle lengthening might alter Sca-1 expression in mMSCs, mMSCs extracted from α7Tg muscle were seeded on laminin (YIGSR peptide) and gelatin (collagen base)-coated flexible membranes and exposed to 10% multiaxial strain for 5 hr. Immediately following strain, transcripts of Sca-1, CD90, and CD29 were notably increased in the presence of laminin, but not gelatin (Fig. 3B and 3C). CD140b was upregulated in response to strain regardless of the substrate, suggesting it is particularly sensitive to mechanical stimuli. Although Rgs5 RNA was expressed, it was not altered in response to strain or substrate.

### mMSCs Upregulate Myogenic Markers Following Mechanical Strain in the Presence of Gelatin

Sca-1^+^CD45^−^ mMSCs extracted from α7Tg muscle 24 hr post-exercise did not spontaneously form myotubes on uncoated cell chamber slides, verifying the absence of satellite cells in our mononuclear fraction (Fig. S2). To determine the full myogenic potential of mMSCs, mMSCs were subjected to mechanical strain in the presence of gelatin and laminin. Myf5 was upregulated following strain and three myogenic markers (Pax3, myogenin and MyoD) were expressed on mMSCs in the presence and absence of strain on *gelatin*. Although the α7 integrin is expressed in myoblasts during development and is considered a myogenic marker, α7 integrin transcription was not upregulated on gelatin and was weakly expressed on laminin in response to strain. The β1 integrin (CD29) was regulated in a similar manner to its heterodimeric partner on laminin (Fig. 3B). In contrast to the results for gelatin, myogenic gene expression was absent in the presence of laminin and was not altered by strain (Fig. 3C).

### Sca-1^+^CD45^−^ MSCs Indirectly Contribute to New Fiber Formation *In Vivo*

**Figure 4:**
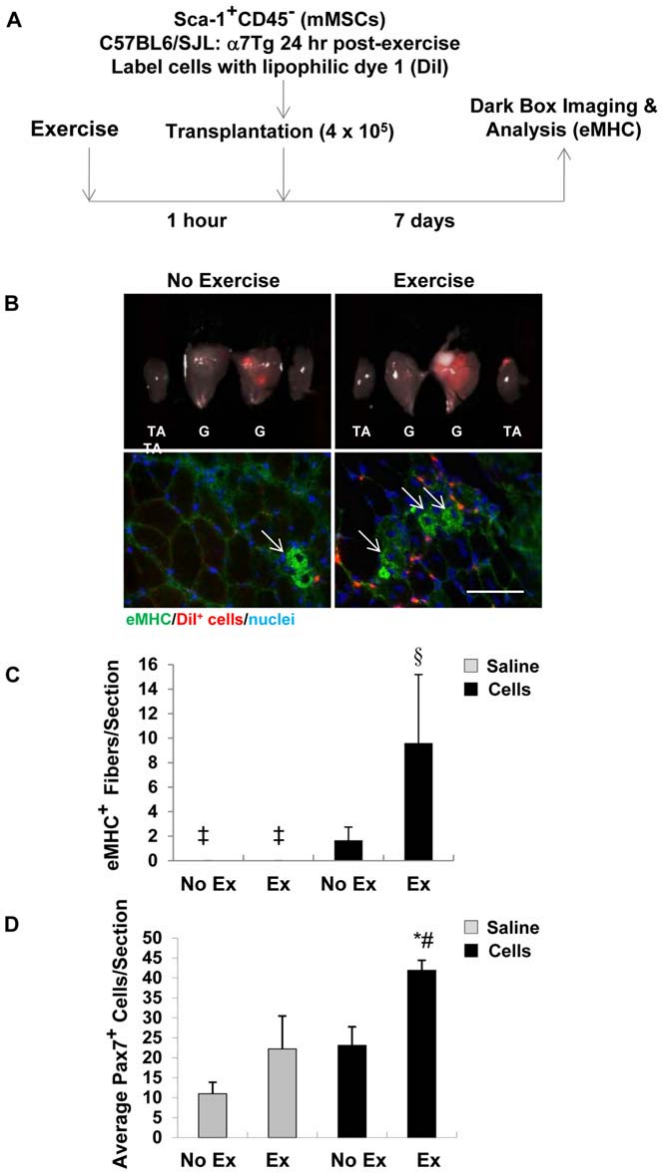
Transplanted mMSCs increase Pax7^+^ cell expansion and myofiber synthesis in pre-exercised skeletal muscle. (*A*) mMSCs were labeled with lipophilic dye (DiI) and transplanted (4×10^5^) into WT recipients that remained sedentary (No Exercise – No Ex) or completed a single bout of eccentric exercise (Exercise - Ex) 1 hr prior to injection. Muscles were dissected 7 days post-exercise to complete *in vivo* dark box imaging and histological analysis for embryonic myosin heavy chain (eMHC). (*B*) (Top) DiI^+^ cells (red) were localized in gastrocnemius-soleus complexes (G) or adjacent tibialis anterior muscles (TA) in saline injected (left set) or cell injected (right set) legs using *in vivo* dark box imaging. (Bottom) New fiber synthesis detected by the presence of eMHC expression (arrows, FITC-green) in close proximity to DiI-labeled cells. (*C*) Quantitation of eMHC^+^ fibers in saline and cell injected muscles. (*D*) Quantitation of Pax7^+^ cells in saline and cell injected muscles. Inset shows image of Pax7 (FITC-green) and DiI (red) co-localization (arrows). ‡ indicates the absence of new fibers in muscle. Scale bar = 5 µm. Data are means ± SEM, n = 3–4/group for *C* and *D*. § P<0.05 vs. all groups, * P<0.01 vs. Saline/No Ex and # P<0.05 vs. Saline/Ex. In *D*, P = 0.051 for Cells/Ex vs. Cells/No Ex.

To assess a direct or indirect role for mMSCs in exercise-induced regeneration and/or growth, cells were extracted from α7Tg muscle 24 hrs post-exercise, labeled with DiI and transplanted into WT recipient muscle (Fig. 4A). A subset of the recipient mice were exercised 1 hr prior to injection to re-establish the exercise microenvironment. Whole tissue localization of DiI-labeled cells in muscle was determined using *in vivo* dark box imaging prior to sectioning (Fig. 4B). Higher fluorescence has detected in all muscles exercised prior to injection with cells compared to mice that did not exercise. No fluorescence was emitted in adjacent uninjected tibialis anterior muscles, nor in saline injected contralateral controls. New fiber synthesis was not detected in saline injected legs, regardless of exercise condition. Conversely, eMHC^+^DiI^−^ fibers were present in close proximity to DiI-labeled cells, with an elevation in eMHC^+^DiI^−^ fibers observed in pre-exercised muscle (P<0.05 Cells/Ex vs. all other groups) (Fig. 4C). Consistent with this observation, Pax7^+^ cells were increased to the greatest extent in skeletal muscle of mice injected with mMSCs following exercise (P<0.01 Cells/Ex vs. Saline/No Ex; P<0.05 Cells/Ex vs. Saline/Ex; P = 0.051 vs. Cells/No Ex) (Fig. 4D). Fusion of DiI^+^ cells with surrounding mature fibers was a rare event.

It is important to note that the α7 integrin transgene is not expressed in mMSCs and cannot account for responses to strain *in vitro* or increased myogenesis following transplantation (Fig. S3).

## Discussion

Satellite cell proliferation is a well-characterized response to eccentric exercise in both humans and animal models, yet minimal information is available regarding how other adult stem cells in muscle respond to lengthening contractions. In this study, we provide the first demonstration that muscle resident (Sca-1^+^CD45^−^) mesenchymal-like stem cells (mMSCs), predominantly pericytes, are increased in muscle in an α7 integrin dependent manner following an acute bout of eccentric exercise. mMSCs maximally appeared in α7BX2 transgenic muscle resistant to injury following eccentric exercise and were rarely present in muscle lacking the α7 integrin, suggesting that factors other than injury or inflammation are primary regulators of mMSC accumulation in skeletal muscle. Isolated mMSCs predominantly characterized as pericytes upregulated MSC markers (Sca-1, CD90) when subjected to mechanical strain in the presence of laminin, providing a mechanistic basis for their appearance in muscle. Transplanted mMSCs stimulated Pax7^+^ cell expansion and facilitated synthesis of new fibers, particularly when cells were injected into muscle subjected to a single bout of exercise. Therefore, mMSCs, the majority pericytes, likely contribute to early events associated with eccentric exercise-induced skeletal muscle adaptations.

We previously established that increased α7 integrin protein is a natural response to injury or strain associated with a single bout of eccentric exercise in WT mice [29], [30], and increased adhesion afforded by the α7 integrin markedly suppresses Evans blue dye fiber uptake, macrophage accumulation and deficits in maximal isometric force production [29]–[31]. We have recently demonstrated that muscle-specific transgenic expression of the α7 integrin accelerates exercise-induced myogenesis and hypertrophy, and that a reduction in macrophage content and/or elevations in Pax7^+^ cells 24 hr post-exercise likely influence new growth observed in the muscles of these mice [31]. We now show that mMSCs are also elevated in α7Tg muscle under resting conditions compared to WT muscle and accumulate to a greater extent in response to exercise. The lack of mMSC presence in α7^−/−^ mice which are susceptible to exercise-induced damage [30] suggests that injury is not essential regulator of interstitial MSC appearance in skeletal muscle. Thus, we are left wondering how the α7 integrin elicits either a direct or indirect effect on mMSC accumulation in muscle.

Our original rationale for examining the mMSC transcriptional response to mechanical strain *in vitro* was to determine the full extent to which muscle-derived Sca-1^+^CD45^−^ cells could directly become myogenic. We found that isolated mMSCs could in fact differentiate toward a muscle lineage in the presence of gelatin (collagen base) following strain, yet this response was inhibited in the presence of laminin. mMSCs upregulated MSC transcripts in the presence of laminin following strain, strongly suggesting that the composition of the extracellular matrix (collagen, laminin) in muscle dictates mMSC fate following exercise. Interestingly, muscle lacking α7 integrin are especially susceptible to exercise-induced injury and fibrosis as indicated by sarcolemmal damage and increased gene expression of metalloproteinase-1 (TIMP-1), thrombospondin 1, tenascin C, procollagen VIII (30, Liu unpublished results). We speculate that increased adhesion provided by the α7 integrin prevents exercise-induced collagen deposition and fibrosis, allowing mMSCs to interact with laminin. Support for this hypothesis can be found in a recent study demonstrating the ability for endurance training to decrease collagen and fibrosis, thereby increasing engraftment of transplanted muscle-derived stem cells (Sca-1^+^CD45^−^) [39]. Future studies will examine collagen and laminin content in muscle cross sections of WT and α7Tg post-exercise to determine if suppression of collagen and/or enrichment of laminin provide the basis for mMSC expansion in muscle.

Whether exogenous laminin can enhance regeneration in response to injury has been the focus of a number of recent papers. Investigators have reported the ability for laminin-111 (LM-111) to optimize satellite cell function during regeneration and ameliorate muscular dystrophy pathology in mice [40], [41]. Myoblast engraftment is dramatically improved in *mdx* mice if co-injected with LM-111 [42]. In addition, laminin can increase myoblast expansion in culture [43]. Thus, it will be important to determine the extent to which adhesion to laminin and/or elastic modulus properties associated with laminin dictate mMSC accumulation and fate in skeletal muscle.

Upregulation of Rgs5 transcripts in α7Tg muscle gene array analysis provided the first clue that the Sca-1^+^CD45^−^ fraction in these mice may represent pericytes [36]. Consistent with ALP^+^CD56^−^ pericytes derived from human skeletal muscle biopsies [3], our cells do not express myogenic markers (Pax7, Myf5, MyoD) or endothelial markers (CD31 and CD34), but do express cell surface markers CD90, CD140b (PDGFRβ), NG2 and CD146 in culture. Hyldahl *et al.* also reported the presence of pericytes in the interstitium of human skeletal muscle, but the total number of cells did not increase *3 hr* post-eccentric exercise [44]. Our preliminary experiments confirm lack of Sca-1^+^CD45^−^ cell expansion in mouse skeletal muscle at 3 hr post-exercise. Thus, future studies should evaluate the presence of pericytes in human muscle at a later time point following eccentric exercise.

Approximately 20–40% of ALP^+^CD56^−^ pericytes derived from human skeletal muscle become myogenic at the onset of differentiation in culture [3]. Injected ALP^+^CD56^−^ pericytes can also upregulate Pax7 and express dystrophin when injected into *scid-mdx* mice. CD31^−^CD45^−^ mesenchymal-like side population cells do not highly engraft or restore dystrophin in immunodeficient NOD/*scid* or *mdx* mice, but can significantly enhance myoblast proliferation, migration and transplantation [4]. In this study we demonstrate that mMSCs *indirectly* stimulate new fiber synthesis in recipient muscle, particularly when the recipient mice are exercised immediately prior to injection. This is not surprising given the plethora of growth factors reportedly secreted by MSCs, including LIF, which can activate satellite cell regeneration and muscle growth [45]. The number of new fibers observed in this study with transplantation is modest and localized to the injection site, yet it is important to keep in mind that we injected a minimal number of cells relative to other studies. The abundance of mMSCs in α7Tg muscle post-exercise provide us the opportunity to complete future detailed studies which will reveal the mechanism by which these cells contribute to eccentric exercise-induced skeletal muscle repair and growth. If mMSCs are a primary source of growth factor, it would be interesting to evaluate the regulation of release following mechanical strain in the presence of different substrates and determine whether mechanically-strained mMSCs can maximize new fiber synthesis and/or fiber growth following transplantation.

mMSCs did not directly contribute to the new fibers observed 7 days following *in vivo* transplantation in this study. However, the ability for mechanical strain to initiate conversion of mMSCs to myogenic cells on a gelatin substrate suggests potential for these cells to directly form new fibers. It will be necessary to examine later time points post-injection (2–4 wk) to determine the full growth potential of mMSCs extracted from α7Tg muscle post-exercise.

It remains controversial whether satellite cells express Sca-1 and the extent to which mMSCs populate the satellite cell niche prior to differentiation [1], [46], [47]. However, we were careful to eliminate potential satellite cell contamination during extraction of Sca-1^+^CD45^−^ cells from α7Tg mice post-exercise. Our cells did not express myogenic markers (Pax7, CD56) before or after transplantation and did not readily differentiate into myotubes in culture. This is consistent with previous studies that report the absence of Sca-1 on satellite cells associated with individual fibers isolated from murine muscle [1]. In addition, Mitchell *et al.* have demonstrated that the majority of quiescent satellite cells are negative for Sca-1 [47]. Therefore, we are confident mMSCs extracted from α7Tg muscle represent a stem cell population distinct from satellite cells.

The α7 integrin transgene is not expressed in Sca-1^+^ cells and is therefore not responsible for dictating mMSC fate in response to strain or facilitating increases in growth following transplantation (Fig. S3). A role for endogenous α7 integrin in regulation of mMSC fate cannot be discounted given the presence of α7 and β1 (CD29) integrin RNA transcripts in Sca-1^+^CD45^−^ cells, especially when subjected to strain on laminin.

Our results demonstrate for the first time that interstitial MSCs, predominantly pericytes, appear in muscle following acute eccentric exercise under the influence of the α7 integrin. *In vitro* experiments using isolated mMSCs suggest that the microenvironment and mechanical strain strongly influence stem cell marker expression, potentially accounting for increased accumulation of these cells in muscle following exercise. Finally, transplantation studies provide evidence that coordinated communication between mMSCs and satellite cells, not satellite cells alone, positively influence regeneration and growth following exercise. This information provides a unique contribution to our basic understanding of the early events in skeletal muscle leading to exercise-induced adaptations. Future studies will focus on the elucidation of niche changes provided by the α7 integrin and/or exercise that influence mMSC fate and identification of factors secreted by mMSCs in response to mechanical strain.

## Materials and Methods

### Transgenic Animals

Protocols for animal use were approved by the Institutional Animal Care and Use Committee (IACUC) of the University of Illinois at Urbana-Champaign (Protocol #10101 and #09179). α7Tg mice (MCK- α7BX2) were produced at the University of Illinois Transgenic Animal Facility as described [29]. α7^−/−^ mice (α7-βgal) were produced at the University of Nevada Transgenic Center as described [48].

### Downhill Running Exercise

Five wk old female WT, α7Tg and α7^−/−^ mice remained at rest (basal conditions) or completed a single bout of downhill running exercise (−20°, 17 m/min, 30 min). Speed on the treadmill (Exer-6M, Columbus Instruments, Columbus, OH) was gradually increased from 10 to 17 m/min during a 2 min warm-up period. Mice were euthanized via carbon dioxide asphyxiation 24 hr post-exercise. The gastrocnemius-soleus complexes were either rapidly dissected and frozen in liquid nitrogen for isolation of RNA, pre-cooled isopentane for immunohistochemistry studies, or incubated in Hank's Buffered Salt Solution (HBSS) for flow cytometry or fluorescence-activated cell sorting (FACS). Evan's blue dye incorporation, an indicator of sarcolemmal damage, was previously assessed using these samples [29], [30].

### Detection of Sca-1^+^ Cell Appearance and Localization by Immunofluorescence

Sca-1 is a cell surface glycoprotein expressed on a variety of cell types, including putative stem/progenitor cell populations within skeletal muscle [49]. Co-localization of the α7B integrin isoform with Sca-1^+^ mononuclear cells was examined in skeletal muscle cross sections. 8-µm-thick sections were fixed in acetone and blocked with PBS containing 5% bovine serum albumin (BSA). Endogenous mouse immunoglobulin was blocked with 70 µg/ml goat anti-mouse monovalent Fab fragments (Jackson ImmunoResearch Laboratories, Inc., West Grove, PA). Sections were incubated with rabbit polyclonal antibodies to the α7B cytoplasmic domain (α7CDB; 1∶500) [50], and subsequently with a rat mononclonal antibody that recognizes Sca-1 (Ly-6A/E) (1∶100) (BD Pharmingen, San Jose, CA). Fluorescein (FITC)-labeled donkey anti-rabbit (1∶100) and rhodamine (TRITC)-labeled donkey anti-rat (1∶100) (Jackson ImmunoResearch) were used to detect α7 integrin and Sca-1, respectively. LIF co-localization with Sca-1^+^ cells was performed with the same methods using LIF antibody (Santa Cruz Biotechnology, N-18, sc-1336) and an appropriate FITC-labeled donkey anti-goat secondary antibody (1∶100). Sections were also analyzed for the presence of Rgs5 (1∶200) (Abcam, Cambridge, MA), CD90 (1∶50) (Thy-1.2, AbD Serotec, Oxford, UK), and NG2 (1∶200) (Millipore, Billerica, MA). Laminin (α2/α4) used to delineate fibers was detected using the 4HB antibody generated in Lydia Sorokin's laboratory (1∶2). Species-specific secondary antibodies were applied at 1∶100–1∶200. For all studies, the primary antibody was eliminated on one section from each staining to confirm specific binding. Slides were mounted using Vectashield containing DAPI (Vector Laboratories, Burlingame, CA) and examined with a Leica DMRXA2 microscope using 20×, 40×, or 63× objectives. Images for all experiments in this study were acquired using a Zeiss AxioCam digital camera and OpenLab software (Zeiss, Thornwood, NY, USA).

### Isolation of Sca-1^+^CD45^−^ Cells from Skeletal Muscle

Twenty-four hr post-exercise, gastrocnemius-soleus complexes muscles were dissected, minced with scissors into small fragments and then digested with a final concentration of 0.2% of collagenase Type 2 (Worthington, Biochemical Corp., Lakewood, NJ), 60 U/ml deoxyribonuclease (Sigma-Aldrich) and 2.5 mM CaCl_2_ in PBS for 45 min at 37°C. Every 15 min the sample was resuspended with a 10 ml pipet to dissociate clumps and release mononuclear cells. Inhibition medium containing 20% FBS, 1% (v/v) penicillin/streptomycin in HBSS was added to the tissue slurries and the suspension was serially filtered through 70 µm and 40 µm nylon meshes (BD Falcon, Franklin Lakes, NJ) two times, respectively. The cell suspension was centrifuged at 450×*g* for 5 min. The cell pellet was resuspended in cold 2% FBS in PBS and counted for staining.

### Cell Sorting, Quantification and Characterization by Flow Cytometry

To characterize the Sca-1^+^CD45^−^ fraction isolated from skeletal muscle, the live mononuclear cell suspension was incubated with anti-mouse CD16/CD32 (1 µg/10^6^ cells) (eBioscience, San Diego, CA) for 15 min on ice in order to block non-specific Fc-mediated interactions. The cells were stained with monoclonal antibodies for Sca-1-PE (600 ng/10^6^ cells) and CD45-APC (300 ng/10^6^ cells) (eBioscience, San Diego, CA) diluted in PBS/2% FBS on ice in the dark for 1 hr. Cells were then washed twice, centrifuged and resuspended in PBS/2% FBS+ 5 µg/ml gentamycin (Gibco, Invitrogen, Carlsbad, CA). FACS was performed using an iCyt Reflection System (Champaign, IL) located at Carle Hospital, Urbana, IL. Hematopoietic cells were excluded by gating out CD45^+^ cells. Sca-1^+^CD45^−^ cells were collected in medium for culture (High glucose Dulbecco's Modified Eagle's Medium [DMEM], 10% FBS, 5 µg/ml gentamycin). Sorted cells were seeded on uncoated tissue culture dishes. Cultures were incubated at 37°C and 5% CO_2_. After three days, nonadherent cells were removed by changing the medium. Subsequently, the medium was replaced every three days or with every passage to a new dish.

Sorted Sca-1^+^CD45^−^ cells were analyzed by flow cytometry following 6 days in culture. The cells were retrieved from the plate with accutase (Innovative Cell Technologies, Inc., San Diego, CA) treatment and resuspended in PBS +2% FBS. The cells were incubated with anti-mouse CD16/CD32 (1 µg/10^6^ cells) (eBioscience, San Diego, CA) for 15 min on ice. The cells were divided into equal aliquots for staining with PE-conjugated monoclonal antibodies for: Sca-1, CD90.2, CD140b and CD31 (eBioscience, San Diego, CA), CD146 and CD34 (Biolegend, San Diego CA), CD56 (Abcam, Cambridge, MA). Indirect staining was performed with anti-NG2 (Millipore, Billerica MA) and a PE-conjugated secondary antibody (Jackson ImmunoResearch). Briefly, cells were incubated with specific antibodies for 1 hr on ice; after washing two times the cells were fixed in 3.7% paraformaldehyde and suspended in a solution of PBS/2% FBS. Flow cytometry was performed with appropriate isotype matched controls on a BD FACSCanto II system and analyzed with BD FACSDiva software (Becton Dickinson, San Jose, CA). Cell analysis was performed on at least 10,000 events for each sample. A primary gate based on physical parameters (forward and side scatter, FSC and SSC, respectively) was set to exclude dead cells or debris. For quantification of MSCs in WT, α7Tg, and α7^−/−^ muscle, the cells were extracted and stained with Sca-1 and CD45 antibodies as described above. The cells were fixed in paraformaldehyde for subsequent flow cytometry analysis with the BD FACSCAnto II system.

### Characterization of Sca-1^+^CD45^−^ Cells by PCR

Total RNA was extracted from cultured cells with QiagenRNeasy Kit according to the manufacturer's protocol (Qiagen, Valencia, CA) and then 800 ng were reverse-transcribed into cDNA by using First Strand cDNA Synthesis Kit for RT-PCR (AMV) (Roche, Indianapolis, IN) using random hexamers, according to the manufacturer's recommendations. The polymerase chain reaction (PCR) for specific target gene was performed with 1 µl of cDNA products. Specific primer sequences and annealing temperatures used for PCR are provided in Table S2. PCR products were separated by 2% agarose gel electrophoresis. WT tissue was used as a positive control for CD45 (data not shown), confirming effective amplification by CD45 primers and lack of expression in Sca-1^+^CD45^−^ cells.

### Quantification of NG2 in Skeletal Muscle

Frozen gastrocnemius/soleus complexes from WT and α7Tg mice were manually ground with a porcelain mortar and pestle chilled in liquid nitrogen. Powdered tissue was homogenized in 10 volumes of an ice-cold buffer containing 20 mM HEPES (pH = 7.4), 2 mM EGTA, 50 mM β-glycerophosphate, 1 mM dithiothreitol, 1 mM Na_3_VO_4_, 1% triton X-100, and 10% glycerol, supplemented with 10 µM leupeptin, 3 mM benzamidine, 5 µM pepstatin A, 1 mM phenylmethylsulfonyl fluoride and 10 µg/ml aprotinin. The homogenates were rotated at 4°C for 1 h, centrifuged at 14,000 *g* for 15 min at 4°C, and supernatant was removed as the detergent-soluble fraction. Protein concentration was determined with the Bradford protein assay using BSA for the standard curve.

Equal amounts of protein (60 µg) were separated by SDS-PAGE using 8% acrylamide gels and transferred to nitrocellulose membranes. Equal protein loading was verified by Ponceau S staining. Membranes were blocked in Tris-buffered saline (pH 7.8) containing 8% BSA and membranes were incubated with NG2 antibody (Millipore, Billerica MA) overnight (1∶1000). HRP-conjugated secondary antibody (1∶2000) (Jackson ImmunoResearch) was applied for 1 hr. Bands were detected using Pierce ECL western blotting substrate (Thermo Scientific, Rockford, IL) and a Bio-Rad ChemiDoc XRS system (Bio-Rad, Hercules, CA).

### Evaluation of Ki67 in Tissue Sections

Muscle sections were stained with anti-Ki67 antibody (1∶100) (Abcam, Cambridge, MA) to determine the average number of proliferating cells. Ki67 staining included a 0.25% Triton X-100 permeabilization step prior to blocking with 10% horse serum. Sections were also co-stained with Sca-1 and Ki67 to determine the average number of proliferating Sca-1^+^ cells. Ki67^+^ cells were counted in a total of 50 fields using the 40× objective.

### *In Vitro* Differentiation of Stem Cells

Sorted Sca-1^+^CD45^−^ cells were seeded and grown on chamber slides until 70–80% confluence in expansion medium. The cultures were shifted to differentiation medium to induce osteogenesis, adipogenesis or chondrogenesis (STEMPRO, Gibco, Invitrogen). The cells were cultured under differentiating conditions and the media was changed every 3 to 4 days following supplier's recommendations. Non-induced controls were kept in expansion medium. After specific periods of cultivation, the cells were fixed with 4% paraformaldehyde solution for 30 min. After fixation, the cells were rinsed with PBS and stained with alizarin red (osteogenesis), oil red (adipogenesis) or alician blue (chondrogenesis) solution and visualized under light microscopy.

### *In Vitro* Mechanical Strain Experiments

mMSCs isolated from WT and α7Tg skeletal muscle 24 hr post-exercise were seeded on laminin or gelatin-coated flexible bottom plates (BioFlex plates, Flexcell International, McKeesport, PA) and incubated at 37°C for 24 to 36 h before applying mechanical strain. Cells maintained under static conditions were used as unstrained controls. The cells were subjected to 10% multiaxial cyclical strain for 5 hr at 1 Hz (9,000 total cycles) using a FX-4000 Flexercell strain unit (BioFlex plates, Flexcell International, McKeesport, PA). Immediately following strain, cells from both the strained samples as well as the unstrained controls, were mechanically removed from plates, briefly centrifuged to a pellet and rinsed in PBS prior to extraction of RNA. Cell surface markers were examined by RT-PCR as described above. This experiment was repeated to ensure consistent results.

### Determination of Myogenic Capacity of mMSCs *In Vitro*

Sca-1^+^CD45^−^ mMSCs isolated from α7Tg muscle 24 hr post-exercise were seeded on 0.1% gelatin coated chamber slides. Differentiation media was added upon confluence and changed every 2 days thereafter. On day 5 of differentiation, myotube morphology was observed and images were obtained.

### Determination of Myogenic Capacity of mMSCs *In Vivo*

Sca-1^+^CD45^−^ mMSCs isolated from α7Tg mice 24 hr post-exercise were cultured for 4 days prior to injection. The cells were not passaged during this time. On the day of injection cells were labeled with lipophilic dye (DiI) and suspended in HBSS. Intramuscular injections at a concentration of 4×10^4^ cells/50 µl HBSS were administered to WT mice (n = 7), and contralateral legs were injected with 50 µl of HBSS. Four of the recipients were exercised 1 hr prior to injection to re-establish the exercise microenvironment (as described above). Seven days following injection the mice were euthanized and gastronemius-soleus complexes were collected. Prior to freezing the muscles were imaged using Maestro *in vivo* dark box imaging technology (Cambridge Research & Instrumentation, Woburn, MA) to examine the localization of DiI-labeled cells in the muscle. eMHC^+^ and Pax7^+^ cells were detected following transplantation of Sca-1^+^CD45^−^ cells by standard immunostaining procedures. Briefly, sections were fixed in acetone for 5 minutes, blocked with 1× PBS/10% horse serum and 70 µg/ml goat anti-mouse monovalent Fab fragments (AffiniPure Fab Fragment Goat Anti-Mouse IgG (H+L), Jackson ImmunoResearch), and incubated with mouse monoclonal 47A (1∶10) antibody (kindly provided by Peter Merrifield, University of Western Ontario, Canada) for 1 hr. Quiescent and activated satellite cells were detected with Pax7 antibody using methods described for eMHC, except that 5% BSA was used for blocking and the antibody (1∶2) (Developmental Studies Hybridoma Bank, Iowa City, Iowa) was applied overnight. Total eMHC positive fibers and Pax7 positive cells were counted in 40 fields using 20× and 40× objectives, respectively (3 sections/sample).

### Detection of the α7 Integrin Transgene in mMSCs

Sca-1^+^CD45^−^ cells were extracted from WT and α7Tg skeletal muscle and subjected to 10% multiaxial strain for 5 hr in the presence of gelatin or laminin. PCR restriction fragment length polymorphism (RFLP) was developed, using *Taq I* restriction enzyme to distinguish between endogenous (mouse) and transgenic (rat) α7 integrin sequences. PCR was performed with 1 µL of cDNA at an annealing temperature of 59°C. Primer sequences used are as follows: F: 5′ AACTGACAGTCACCAACCTGCC 3′ and R: 5′ CCTTGATTGGAGACCGTGACC 3′. 7 µl of the PCR product was digested with *Taq I* for at least 2 hr and the fragments were resolved in 2% agarose gels stained with ethidium bromide.

### Statistical Analysis

All averaged data are presented as the means ± SE. Two-way ANOVA was completed to determine if an interaction effect was observed for group and time. Main effects were analyzed by one-way ANOVA, followed by Tukey's (Sigma Stat®) or LSD (SPSS, version 16) post hoc analysis. For evaluation of Sca-1^−^CD45^+^ (hematopoietic) cell content in muscle, unpaired t-tests were used to determine significant differences. Differences were considered significant at *P*<0.05.

## Supporting Information

Figure S1 **Examination of pericyte markers in α7Tg skeletal muscle 24 hr post-exercise.** (*A*) Co-localization of stem cell antigen-1 (Sca-1) (TRITC-red) and α7 integrin (FITC-green) in the interstitium at higher magnification (63×). Scale bar = 5 µm. (*B*) mMSC morphology and differentiation capacity. (a) morphology of Sca-1^+^CD45^−^ cells isolated from α7Tg muscle 24hPE after 6 days in culture; scale bar = 20 µm (b) alizarin red staining of Sca-1^+^CD45^−^ cells in osteogenic media; Scale bar = 10 µm (c) alician blue staining of Sca-1^+^CD45^−^ cells in chondrogenic media; Scale bar = 10 µm (d) oil red staining of Sca-1^+^CD45^−^ cells in adipogenic media; Scale bar = 10 µm.
(PPTX)

Figure S2 **mMSCs do not spontaneously differentiate into myotubes.** Differentiation capabilities of Sca-1^+^CD45^−^ cells isolated from α7Tg mice post-exercise (mMSCs) compared to primary muscle control cells. Scale bar = 20 µm.
(PPTX)

Figure S3 **mMSCs do not express the α7 integrin transgene.** Sca-1^+^CD45^−^ cells were extracted from wild type (WT) and α7Tg (Tg) skeletal muscle 24 hr post-exercise and subjected to 10% multiaxial strain for 5 hr in the presence of gelatin and laminin. A PCR-restriction fragment length polymorphism protocol was used to distinguish endogenous and transgenic α7 integrin sequences. Primary cells from WT mouse skeletal muscle and α7 integrin rat cDNA were used as negative and positive controls (arrow).
(PPTX)

Table S1 **Flow cytometry analysis of Sca-1 and CD45.** Data are means ± SEM, n = 4–6/group. * P<0.05.
(PPTX)

Table S2 **Primer sequence information.**
(PPTX)

## References

[1] Asakura A, Seale P, Girgis-Gabardo A, Rudnicki MA (2002). Myogenic specification of side population cells in skeletal muscle.. J Cell Biol.

[2] Sampaolesi M, Blot S, D'Antona G, Granger N, Tonlorenzi R (2006). Mesoangioblast stem cells ameliorate muscle function in dystrophic dogs.. Nature.

[3] Dellavalle A, Sampaolesi M, Tonlorenzi R, Tagliafico E, Sacchetti B (2007). Pericytes of human skeletal muscle are myogenic precursors distinct from satellite cells.. Nat Cell Biol.

[4] Motohashi N, Uezumi A, Yada E, Fukada S, Fukushima K (2008). Muscle CD31 (-) CD45 (-) side population cells promote muscle regeneration by stimulating proliferation and migration of myoblasts.. Am J Pathol.

[5] Qu Z, Balkir L, van Deutekom JC, Robbins PD, Pruchnic R (1998). Development of approaches to improve cell survival in myoblast transfer therapy.. J Cell Biol.

[6] Qu-Petersen Z, Deasy B, Jankowski R, Ikezawa M, Cummins J (2002). Identification of a novel population of muscle stem cells in mice: potential for muscle regeneration.. J Biol Chem.

[7] Gussoni E, Soneoka Y, Strickland CD, Buzney EA, Khan MK (1999). Dystrophin expression in the mdx mouse restored by stem cell transplantation.. Nature.

[8] Montanaro F, Liadaki K, Schienda J, Flint A, Gussoni E (2004). Demystifying SP cell purification: viability, yield, and phenotype are defined by isolation parameters.. Exp Cell Res.

[9] Uezumi A, Ojima K, Fukada S, Ikemoto M, Masuda S (2006). Functional heterogeneity of side population cells in skeletal muscle.. Biochem Biophys Res Commun.

[10] Mitchell KJ, Pannerec A, Cadot B, Parlakian A, Besson V (2010). Identification and characterization of a non-satellite cell muscle resident progenitor during postnatal development.. Nat Cell Biol.

[11] Tamaki T, Akatsuka A, Yoshimura S, Roy RR, Edgerton VR (2002). New fiber formation in the interstitial spaces of rat skeletal muscle during postnatal growth.. J Histochem Cytochem.

[12] Torrente Y, Tremblay JP, Pisati F, Belicchi M, Rossi B (2001). Intraarterial injection of muscle-derived CD34(+)Sca-1(+) stem cells restores dystrophin in mdx mice.. J Cell Biol.

[13] Snijders T, Verdijk LB, van Loon LJC (2009). The impact of sarcopenia and exercise training on skeletal muscle satellite cells.. Ageing Res Rev.

[14] Verdijk LB, Gleeson BG, Jonkers RAM, Meijer K, Savelberg HH (2009). Skeletal muscle hypertrophy following resistance training is accompanied by a fiber type-specific increase in satellite cell content in elderly men.. J Gerontol A Biol Sci Med Sci.

[15] Blaauw B, Canato M, Agatea L, Toniolo L, Mammucari C (2009). Inducible activation of Akt increases skeletal muscle mass and force without satellite cell activation.. FASEB J.

[16] Bruusgaard JC, Johansen IB, Egner IM, Rana ZA, Gundersen K (2010). Myonuclei acquired by overload exercise preceed hypertrophy and are not lost on detraining.. Proc Natl Acad Sci USA.

[17] O'Connor RS, Pavlath GK (2007). Point:Counterpoint: Satellite cell addition is/is not obligatory for skeletal muscle hypertrophy.. J Appl Physiol.

[18] Petrella JK, Kim JS, Mayhew DL, Cross JM, Bamman MM (2008). Potent myofiber hypertrophy during resistance training in humans is associated with satellite cell-mediated myonuclear addition: a cluster analysis.. J Appl Physiol.

[19] Kadi F, Thornell LE (1999). Training affects myosin heavy chain phenotype in the trapezius muscle of women.. Histochem Cell Biol.

[20] LaBarge M, Blau HM (2002). Biological progression from adult bone marrow to mononucleate muscle stem cell to multinucleate muscle fiber in response to injury.. Cell.

[21] Palermo AT, Labarge MA, Doyonnas R, Pomerantz J, Blau HM (2005). Bone marrow contribution to skeletal muscle: a physiological response to stress.. Dev Biol.

[22] Parise G, O'Reilly CE, Rudnicki MA (2006). Molecular regulation of myogenic progenitor populations.. Appl Physiol Nutr Metab.

[23] Hynes RO (2002). Integrins: bidirectional, allosteric signaling machines.. Cell.

[24] Paul AC, Sheard PW, Kaufman SJ, Duxson MJ (2002). Localization of alpha 7 integrins and dystrophin suggests potential for both lateral and longitudinal transmission of tension in large mammalian muscles.. Cell Tissue Res.

[25] Bao ZZ, Lakonishok M, Kaufman S, Horwitz AF (1993). α7β1 integrin is a component of the myotendinous junction on skeletal muscle.. J Cell Sci.

[26] Martin PT, Kaufman SJ, Kramer RH, Sanes JR (1996). Synaptic integrins: Selective association of the α1 and α7A, and α7B subunits with the neuromuscular junction.. Dev Biol.

[27] Mayer U, Saher G, Fassler R, Bornemann A, Echtermeyer F (1997). Absence of integrin alpha 7 causes a novel form of muscular dystrophy.. Nat Genet.

[28] Hayashi YK, Chou FL, Engvall E, Ogawa M, Matsuda C (1998). Mutations in the integrin alpha7 gene cause congenital myopathy.. Nat Genet.

[29] Boppart MD, Burkin DJ, Kaufman SJ (2006). α7β1-Integrin regulates mechanotransduction and prevents skeletal muscle injury.. Am J Physiol Cell Physiol.

[30] Boppart MD, Volker SE, Alexander N, Burkin DJ, Kaufman SJ (2008). Exercise promotes α7 gene transcription and protection of skeletal muscle.. Am J Physiol Regul Integr Comp Physiol.

[31] Lueders TN, Zou K, Huntsman HD, Meador B, Mahmassani Z (2011). The α7β1 integrin accelerates fiber hypertrophy and myogenesis following a single bout of eccentric exercise.. Am J Physiol Cell Physiol.

[32] Zou K, Meador BM, Johnson B, Huntsman HD, Mahmassani Z (2011). The α7β1 integrin increases muscle hypertrophy following multiple bouts of eccentric exercise.. J Appl Physiol.

[33] Lolmede K, Campana L, Vezzoli M, Bosurgi L, Tonlorenzi R (2009). Inflammatory and alternatively activated human macrophages attract vessel-associated stem cells, relying on separate HMGB1- and MMP-9-dependent pathways.. J Leukoc Biol.

[34] Whitney MJ, Lee A, Ylostalo J, Zeitouni S, Tucker A (2009). Leukemia inhibitory factor secretion is a predictor and indicator of early progenitor status in adult bone marrow stromal cells.. Tissue Eng Part A.

[35] Gang EJ, Bosnakovski D, Simsek T, To K, Perlingeiro RC (2008). Pax3 activation promotes the differentiation of mesenchymal stem cells toward the myogenic lineage.. Exp Cell Res.

[36] Liu J, Burkin DJ, Kaufman SJ (2008). Increasing α7β1-integrin promotes muscle cell proliferation, adhesion, and resistance to apoptosis without changing gene expression.. Am J Physiol Cell Physiol.

[37] Yutoku M, Grossberg AL, Pressman D (1974). A cell surface antigenic determinant present on mouse plasmacytes and only about half of mouse thymocytes.. J Immunol.

[38] Kadafar KA, Yi L, Ahmad Y, So L, Rossi F (2009). Sca-1 expression is required for efficient remodeling of the extracellular matrix during skeletal muscle regeneration.. Dev Biol.

[39] Ambrosio F, Ferrari RJ, Distefano G, Plassmeyer JM, Carvell GE (2010). The synergistic effect of treadmill running on stem cell transplantation to heal injured skeletal muscle.. Tissue Eng Part A.

[40] Rooney JE, Gurpur PB, Burkin DJ (2009). Laminin-111 protein therapy prevents muscle disease in the mdx mouse model for Duchenne muscular dystrophy.. Proc Natl Acad Sci.

[41] Rooney JE, Gurpur PB, Yablonka-Reuveni Z, Burkin DJ (2009). Laminin-111 restores regenerative capacity in a mouse model for alpha7 integrin congenital myopathy.. Am J Pathol.

[42] Goudenege S, Lamarre Y, Dumont N, Rousseau J, Frenette J (2010). Laminin-111: a potential therapeutic agent for Duchenne muscular dystrophy.. Mol Ther.

[43] Kino-oka M, Chowdhury SR, Muneyuki Y, Manabe M, Saito A (2009). Automating the expansion process of human skeletal muscle myoblasts with suppression of myotube formation.. Tissue Eng Part C Methods.

[44] Hyldahl RD, Xin L, Hubal MJ, Moeckel-Cole S, Chipkin S (2011). Activation of nuclear factor-κB following muscle eccentric contractions in humans is localized primarily to skeletal muscle-residing pericytes.. FASEB J.

[45] Spagenburg EE, Booth FW (2002). Multiple signaling pathways mediate LIF-induced skeletal muscle satellite cell proliferation.. Am J Physiol Cell Physiol.

[46] Zammit P, Beauchamp J (2001). The skeletal muscle satellite cell: stem cell or son of stem cell?. Differentiation.

[47] Mitchell PO, Mills T, O'Connor RS, Kline ER, Graubert T (2005). Sca-1 negatively regulates proliferation and differentiation of muscle cells.. Dev Biol.

[48] Flintoff-Dye NL, Welser J, Rooney J, Scowen P, Tamowski S (2005). Role for the α7β1 integrin in vascular development and integrity.. Dev Dyn.

[49] Holmes C, Stanford WL (2007). Concise review: stem cell antigen-1: expression, function, and enigma.. Stem Cells.

[50] Song WK, Wang W, Sato H, Bielser DA, Kaufman SJ (1993). Expression of α7 integrin cytoplasmic domains during skeletal muscle development: Alternate forms, conformational change, and homologies with serine/threonine kinases and tyrosine phosphatases.. J Cell Sci.

