# Supplementary figures and images for: Eccentric Exercise Facilitates Mesenchymal Stem Cell Appearance in Skeletal Muscle
Source: PLoS One. 2012 Jan 11;7(1):e29760. doi: 10.1371/journal.pone.0029760 (PMC3256189; doi:10.1371/journal.pone.0029760)

## Slide 1
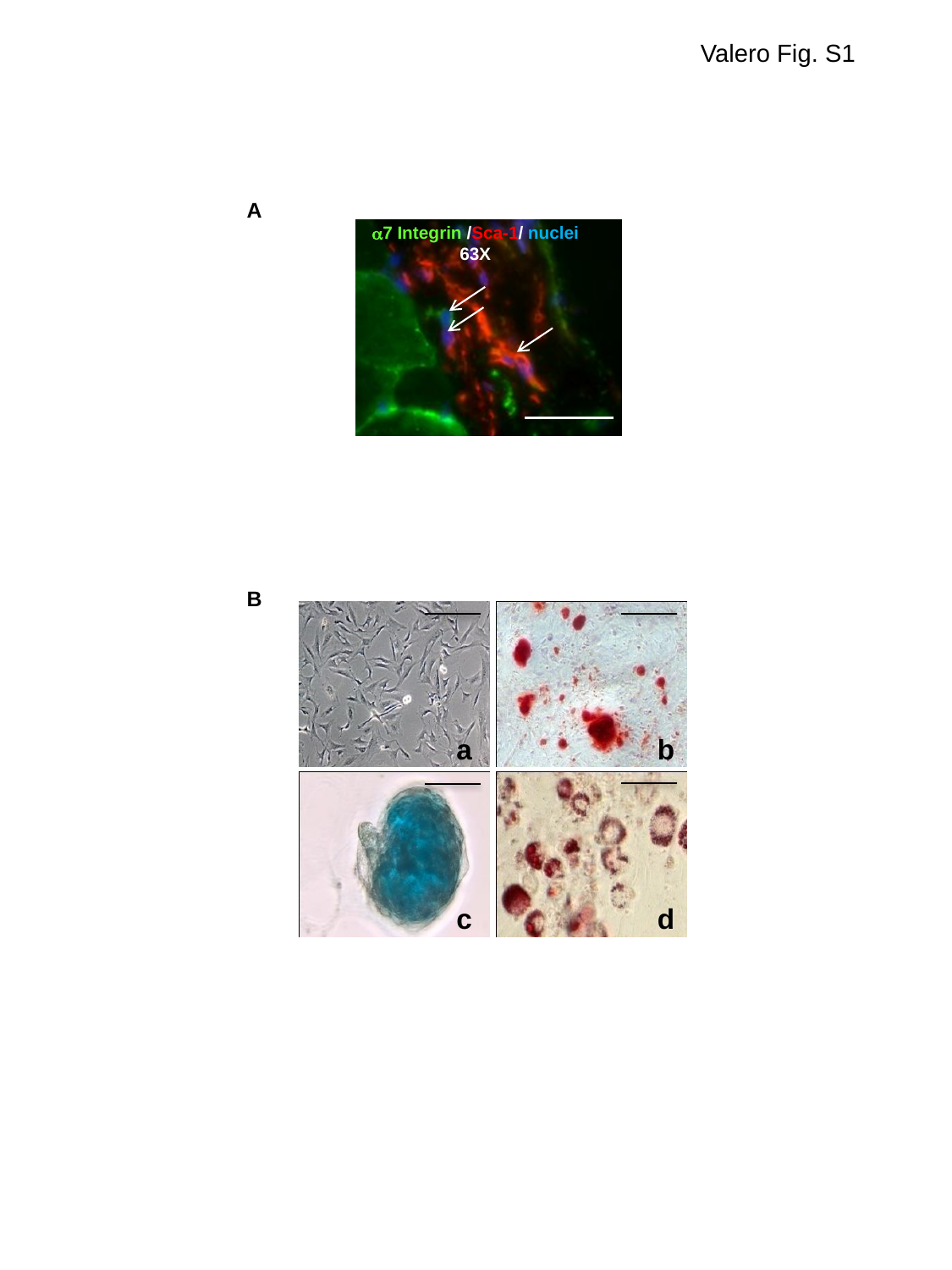

Valero Fig. S1
A
α7 Integrin /Sca-1/ nuclei
63X
B
a
b
c
d

Supplement: Figure S1 — Examination of pericyte markers in α7Tg skeletal muscle 24 hr post-exercise. (A) Co-localization of stem cell antigen-1 (Sca-1) (TRITC-red) and α7 integrin (FITC-green) in the interstitium at higher magnification (63×). Scale bar = 5 µm. (B) mMSC morphology and differentiation capacity. (a) morphology of Sca-1+CD45− cells isolated from α7Tg muscle 24hPE after 6 days in culture; scale bar = 20 µm (b) alizarin red staining of Sca-1+CD45− cells in osteogenic media; Scale bar = 10 µm (c) alician blue staining of Sca-1+CD45− cells in chondrogenic media; Scale bar = 10 µm (d) oil red staining of Sca-1+CD45− cells in adipogenic media; Scale bar = 10 µm. (PPTX) [file pone.0029760.s001.pptx]

## Slide 1
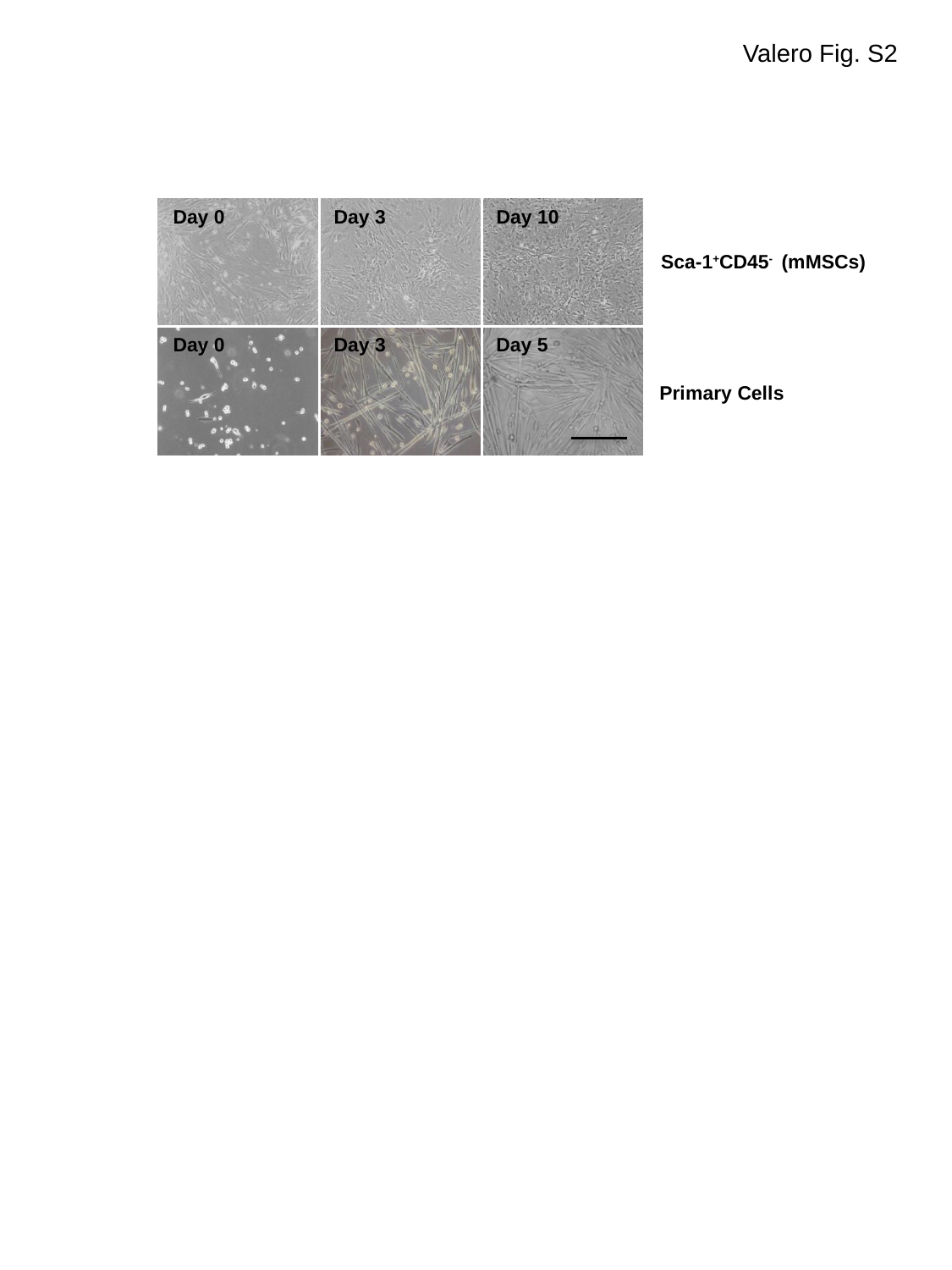

Valero Fig. S2
Day 0
Day 3
Day 10
Day 0
Day 3
Day 5
Sca-1+CD45- (mMSCs)
Primary Cells

Supplement: Figure S2 — mMSCs do not spontaneously differentiate into myotubes. Differentiation capabilities of Sca-1+CD45− cells isolated from α7Tg mice post-exercise (mMSCs) compared to primary muscle control cells. Scale bar = 20 µm. (PPTX) [file pone.0029760.s002.pptx]

## Slide 1
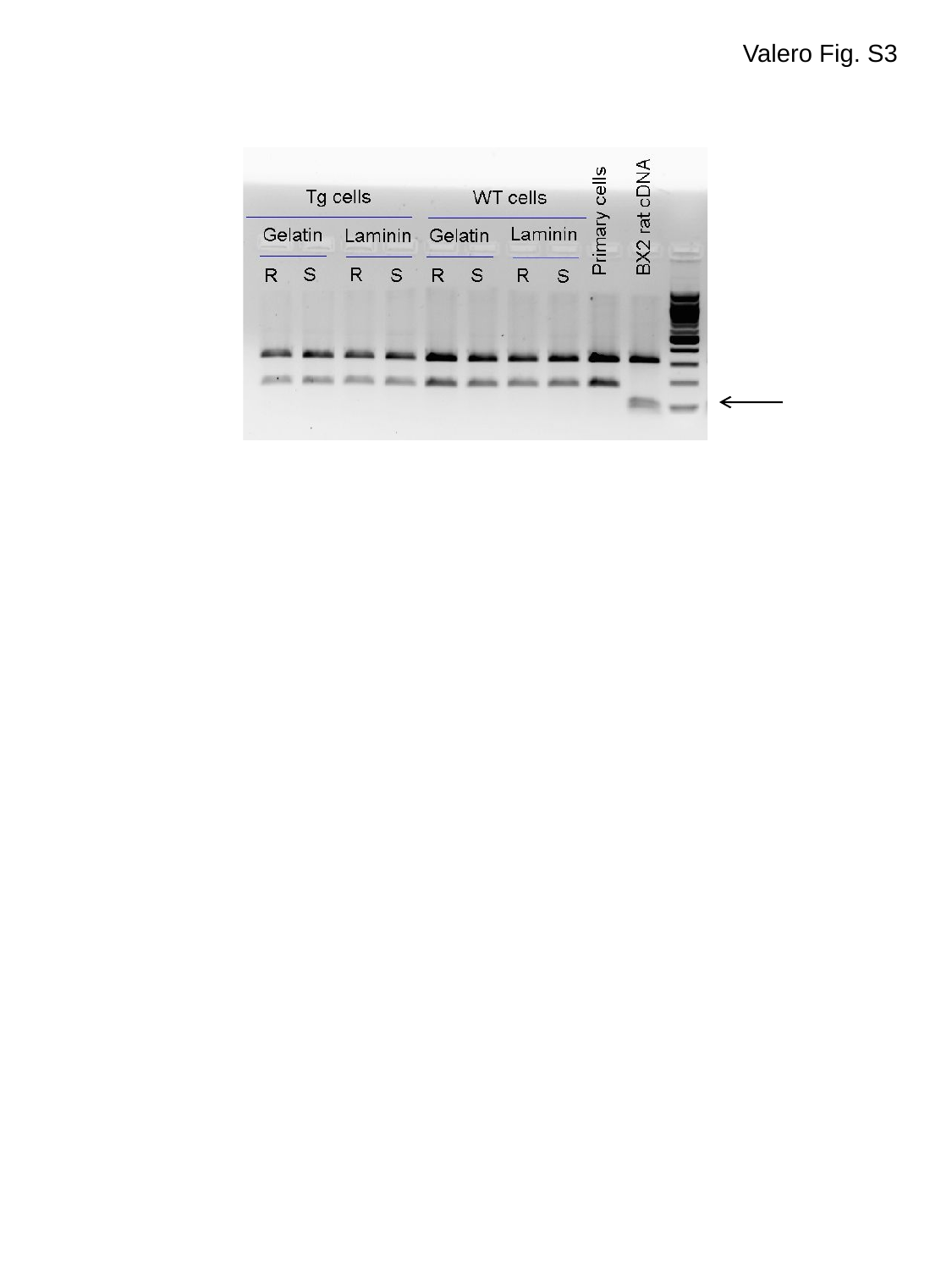

Valero Fig. S3

Supplement: Figure S3 — mMSCs do not express the α7 integrin transgene. Sca-1+CD45− cells were extracted from wild type (WT) and α7Tg (Tg) skeletal muscle 24 hr post-exercise and subjected to 10% multiaxial strain for 5 hr in the presence of gelatin and laminin. A PCR-restriction fragment length polymorphism protocol was used to distinguish endogenous and transgenic α7 integrin sequences. Primary cells from WT mouse skeletal muscle and α7 integrin rat cDNA were used as negative and positive controls (arrow). (PPTX) [file pone.0029760.s003.pptx]

## Slide 1
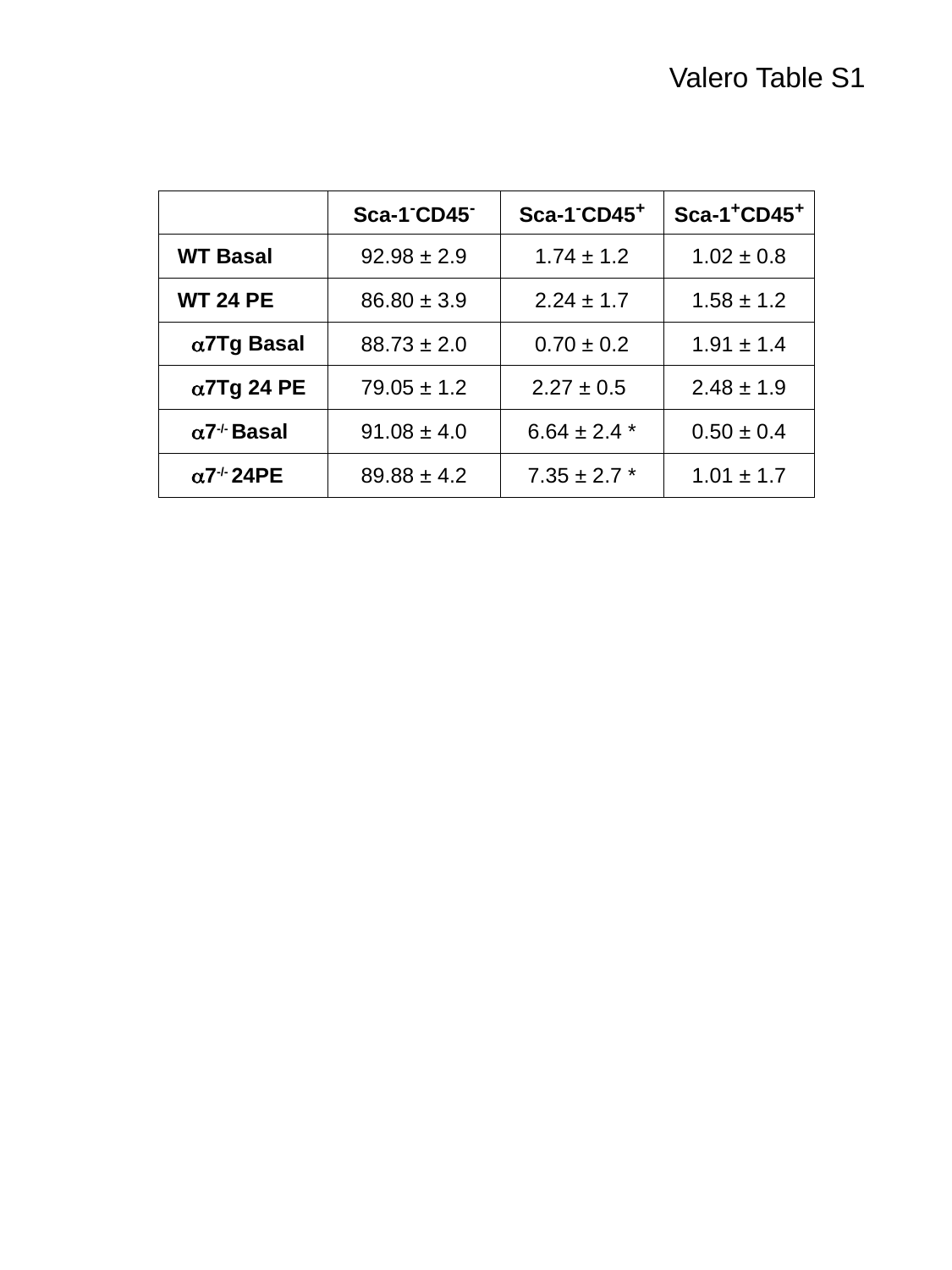

Valero Table S1
| | Sca-1-CD45- | Sca-1-CD45+ | Sca-1+CD45+ |
| --- | --- | --- | --- |
| WT Basal | 92.98 ± 2.9 | 1.74 ± 1.2 | 1.02 ± 0.8 |
| WT 24 PE | 86.80 ± 3.9 | 2.24 ± 1.7 | 1.58 ± 1.2 |
| α7Tg Basal | 88.73 ± 2.0 | 0.70 ± 0.2 | 1.91 ± 1.4 |
| α7Tg 24 PE | 79.05 ± 1.2 | 2.27 ± 0.5 | 2.48 ± 1.9 |
| α7-/- Basal | 91.08 ± 4.0 | 6.64 ± 2.4 \* | 0.50 ± 0.4 |
| α7-/- 24PE | 89.88 ± 4.2 | 7.35 ± 2.7 \* | 1.01 ± 1.7 |

Supplement: Table S1 — Flow cytometry analysis of Sca-1 and CD45. Data are means ± SEM, n = 4–6/group. * P<0.05. (PPTX) [file pone.0029760.s004.pptx]
