# Supplementary material for: Eccentric Exercise Facilitates Mesenchymal Stem Cell Appearance in Skeletal Muscle
Source: PLoS One. 2012 Jan 11;7(1):e29760. doi: 10.1371/journal.pone.0029760 (PMC3256189; doi:10.1371/journal.pone.0029760)
Supplement: Table S2 — Primer sequence information. (PPTX) [file pone.0029760.s005.pptx]

## Slide 1
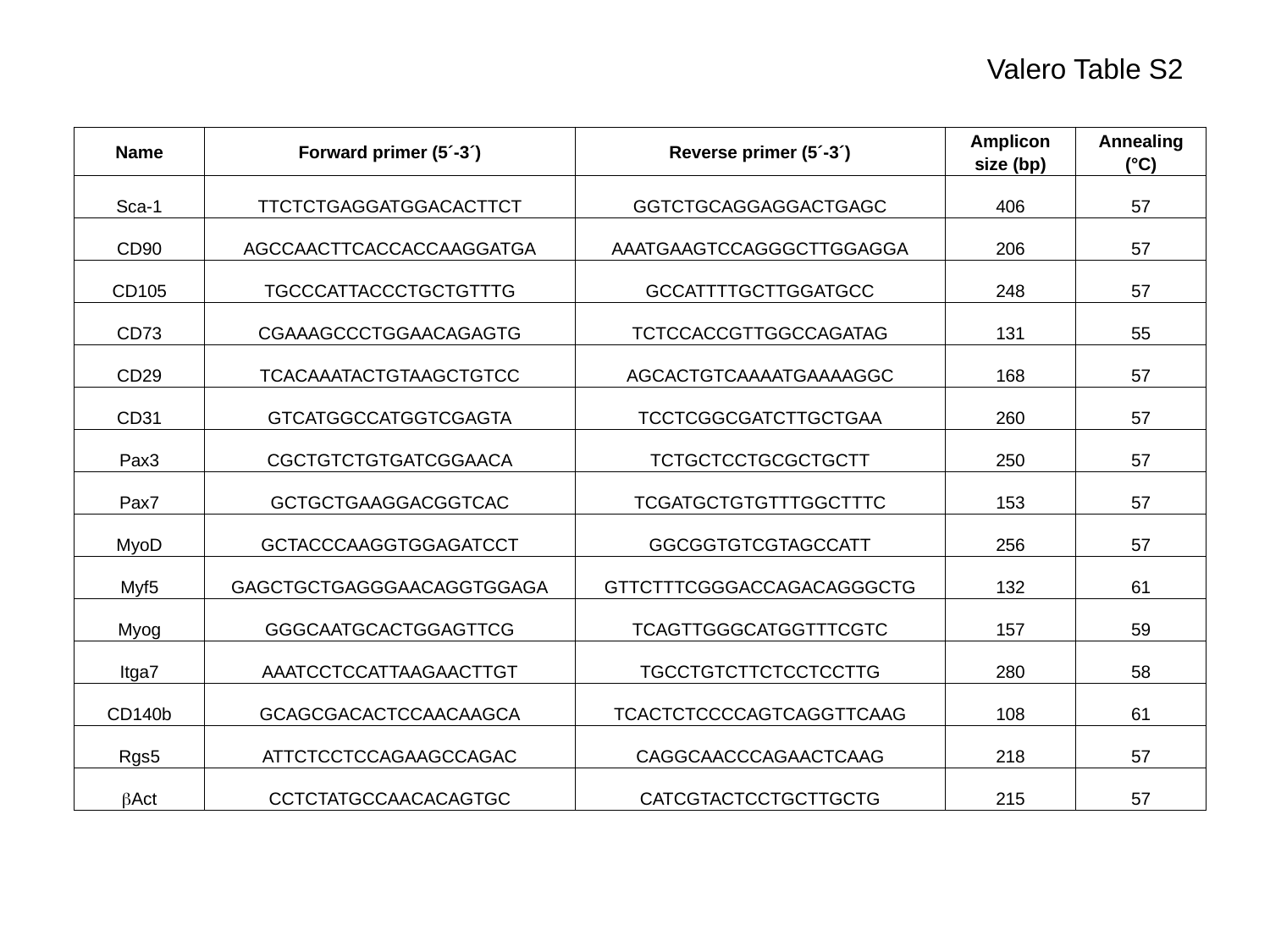

Valero Table S2
| Name | Forward primer (5´-3´) | Reverse primer (5´-3´) | Amplicon size (bp) | Annealing (°C) |
| --- | --- | --- | --- | --- |
| Sca-1 | TTCTCTGAGGATGGACACTTCT | GGTCTGCAGGAGGACTGAGC | 406 | 57 |
| CD90 | AGCCAACTTCACCACCAAGGATGA | AAATGAAGTCCAGGGCTTGGAGGA | 206 | 57 |
| CD105 | TGCCCATTACCCTGCTGTTTG | GCCATTTTGCTTGGATGCC | 248 | 57 |
| CD73 | CGAAAGCCCTGGAACAGAGTG | TCTCCACCGTTGGCCAGATAG | 131 | 55 |
| CD29 | TCACAAATACTGTAAGCTGTCC | AGCACTGTCAAAATGAAAAGGC | 168 | 57 |
| CD31 | GTCATGGCCATGGTCGAGTA | TCCTCGGCGATCTTGCTGAA | 260 | 57 |
| Pax3 | CGCTGTCTGTGATCGGAACA | TCTGCTCCTGCGCTGCTT | 250 | 57 |
| Pax7 | GCTGCTGAAGGACGGTCAC | TCGATGCTGTGTTTGGCTTTC | 153 | 57 |
| MyoD | GCTACCCAAGGTGGAGATCCT | GGCGGTGTCGTAGCCATT | 256 | 57 |
| Myf5 | GAGCTGCTGAGGGAACAGGTGGAGA | GTTCTTTCGGGACCAGACAGGGCTG | 132 | 61 |
| Myog | GGGCAATGCACTGGAGTTCG | TCAGTTGGGCATGGTTTCGTC | 157 | 59 |
| Itga7 | AAATCCTCCATTAAGAACTTGT | TGCCTGTCTTCTCCTCCTTG | 280 | 58 |
| CD140b | GCAGCGACACTCCAACAAGCA | TCACTCTCCCCAGTCAGGTTCAAG | 108 | 61 |
| Rgs5 | ATTCTCCTCCAGAAGCCAGAC | CAGGCAACCCAGAACTCAAG | 218 | 57 |
| βAct | CCTCTATGCCAACACAGTGC | CATCGTACTCCTGCTTGCTG | 215 | 57 |
